# Empathy and Psychosocial Adjustment in Tibetan and Han Adolescents: A Person-Centered Approach

**DOI:** 10.3389/fpsyg.2019.01896

**Published:** 2019-08-13

**Authors:** Chunhua Ma, Yongfeng Ma, Youpeng Wang, Xiaoyu Lan

**Affiliations:** ^1^College of Educational Science and Technology, Northwest Minzu University, Lanzhou, China; ^2^Department of Petrochemical Engineering, Lanzhou Petrochemical College of Vocational Technology, Lanzhou, China; ^3^Faculty of Psychology, Beijing Normal University, Beijing, China; ^4^Department of Developmental Psychology and Socialization, University of Padua, Padua, Italy

**Keywords:** affective empathy, cognitive empathy, Tibetan adolescent, Han adolescent, psychosocial adjustment

## Abstract

Although prior research has shown potential academic difficulties for Tibetan adolescents when coping with the mainstream Han culture, little is known about their psychosocial adjustment. Adopting a person-centered approach, the current study explores psychosocial adjustment profiles based on internalizing indicators (i.e., depression, loneliness, life satisfaction, positive affect, and negative affect) and externalizing indicators (i.e., prosocial behavior and antisocial behavior). Moreover, guided by the empathy theory, this study also examines the direct and interactive effects of empathy (affective and cognitive empathy) and ethnicity (Tibetan vs. Han adolescents) on psychosocial adjustment profiles. A total of 306 Tibetan adolescents (66.3% girls) and 321 Han adolescents (55.1% girls) were involved in this study, and participants were asked to fill in a set of self-report questionnaires. A latent profile analysis revealed five psychosocial adjustment profiles: adaptive, maladaptive, externalizing, internalizing, and moderate. Furthermore, a multiple multinomial analysis showed Han adolescents were more likely than Tibetan adolescents to be a member of adaptive and moderate profiles than of the externalizing profile. Individuals with low affective empathy and high cognitive empathy were prone to be adequate in terms of psychosocial functioning, and the effects of low affective empathy and high cognitive empathy on psychosocial functioning were highlighted in Han adolescents only.

## Introduction

With the interdependence of the economy and the intermingling of cultures, inter-cultural communications are expanding dramatically within mainland China, which has triggered a mounting interest in understanding how ethnic minorities cope with the mainstream culture of China (the Han ethnic group; Yong, 2013). As a diverse cultural entity, there are 55 ethnic minority groups residing in China, and some of them still maintain their traditions, cultural orientations, and even languages (Poston and Shu, 2006). The Tibetan culture, one of the biggest of these ethnic minorities (approximately 7 million Tibetans residing in mainland China; National Bureau of Statistics, 2010), has distinct ethnic characteristics, including physique, geography, language, and religion (Lan et al., 2019a). Due to these significant differences from the mainstream culture of China, a burgeoning body of research (e.g., Yong, 2013) has shown that Tibetan youth experience more difficulties (e.g., poor academic performance) than their Han peers because of language barriers and insufficient education. However, scant attention has been paid to psychosocial adjustment among Tibetan youth in the process of acculturation. This gap is especially glaring for adolescence. As documented by prior research (Larson and Lampman-Petraitis, 1989; Radloff, 1991), adolescents in the midst of psychological, physical, and social transformations experience more frequent and intense socioemotional changes than children and adults. Therefore, it is critical to investigate Tibetan adolescents’ psychosocial adjustment, as compared with their peers in the mainstream context (i.e., Han adolescents). Likewise, exploring the potential correlates of their psychosocial adjustment may offer some insights into designing some school activities to facilitate their positive psychosocial adjustment in the process of acculturation.

In particular, the current study aims to examine psychosocial adjustment in Tibetan and Han adolescents and explores the direct and interactive associations of empathy and ethnicity with psychosocial adjustment. In the next sections, we conducted a literature review about the main study variables (i.e., psychosocial adjustment and empathy) in the current study.

### Psychosocial Adjustment in Adolescence

*Psychosocial adjustment* is a multidimensional construct that entails a set of psychological and social characteristics (Leung et al., 2010; Otterpohl and Wild, 2015). With regard to psychosocial adjustment differences between Tibetan and Han adolescents, prior research has documented that Tibetan adolescents show a higher prevalence of internet addiction than Han counterparts (Lu et al., 2018). Moreover, the smoking of Tibetan adolescents is more intense than that of Han peers (Chen L. et al., 2014). In view of these findings and potential acculturation difficulties of Tibetan youth, we proposed that Tibetan adolescents might be more vulnerable, in terms of psychosocial functioning, than Han adolescents.

Despite such findings, numerous studies have relied on a variable-centered approach, which makes it difficult to examine the distinct combinations of psychosocial outcomes. Thus, the current study adopts a person-centered approach to identify potential psychosocial adjustment profiles in Tibetan and Han adolescents, and may have distinct implications for Tibetan and Han adolescents when characterizing a particular group of individuals belonging to a specific psychosocial type. With regard to psychosocial profiles, Zhao et al. (2019) report three profiles: an adequate adaptation profile, an internalizing problem profile, and an externalizing problem profile based on internalizing indicators (i.e., depressive symptoms, loneliness, subjective happiness, and life satisfaction) and externalizing indicators (i.e., rule-breaking behavior, aggressive behavior, and prosocial behavior) in a sample of Chinese left-behind adolescents. Informed by this study, we operationalized psychosocial adjustment based on the combinations of internalizing (i.e., depression, loneliness, life satisfaction, positive affect, and negative affect) and externalizing indicators (i.e., prosocial behavior and antisocial behavior). First, both positive and negative indicators can allow us to understand whether these psychosocial outcomes can coexist in Tibetan and Han adolescents. Second, both emotional and behavioral outcomes can help us to examine whether any discrepancies exist between them in Tibetan and Han adolescents.

### The Role of Empathy

In light of the potential vulnerabilities of Tibetan adolescents, exploring the facilitators of their psychosocial adjustment deserves further attention. Guided by a theoretical framework of bilingual-bicultural adaptation (Triandis, 1980), the major variables that determine how a minority person will adjust in a majority society are cognitive and affective characteristics. The variable selection for this study was built on the empathy theory, suggesting that empathy plays a central role in shaping human behavior and psychosocial functioning (Miller and Eisenberg, 1988; Smith, 2006). *Empathy* involves sensitivity to and an understanding of the mental states of others (Davis, 1983). In the literature, empathy has been regarded as either an affective or a cognitive characteristic. The former has been regarded as the ability to experience and share the emotions of others (Mehrabian and Epstein, 1972), and often results in empathic concern, which involves feelings of sorrow or concern for others (Eisenberg, 2000). The latter, which can be described as perspective-taking, refers to the ability to understand others’ emotions (Davis, 1983). Within the theoretical approach of empathy (Smith, 2006), affective empathy and cognitive empathy are two separate, complementary systems in shaping human behavior. Based on previous studies, affective empathy is positively related to internalizing problems (Tone and Tully, 2014), whereas both affective and cognitive empathy are inversely associated with conduct problems among inpatient adolescents from North America (Gambin and Sharp, 2016). For Chinese adolescents, research has shown that cognitive empathy is negatively associated with indirect aggression, and affective empathy might not be a sufficient predictor for aggression (Li et al., 2015). Given these inconsistent findings about the associations of affective empathy and cognitive empathy with psychosocial functioning, further investigation is warranted to ascertain these conflicts.

Furthermore, embedded in Buddhism, Tibetan society attaches special importance to meditation, mindfulness, and spirituality; as a result, a Tibetan is usually inclined to report high levels of empathy that is related to Buddhism (O’Connor et al., 2015). Therefore, it may be possible that the association between empathy and psychosocial adjustment is moderated by ethnicity (Tibetan vs. Han adolescents) because of distinct spiritual beliefs across the two groups. In accordance with theoretical and empirical considerations reviewed above, this study investigates the direct and interactive effects of empathy (i.e., cognitive and affective empathy) and ethnicity (Tibetan vs. Han adolescents) on potential psychosocial adjustment profiles.

### The Present Study

In sum, the present study has two main goals: (a) to explore potential psychosocial adjustment profiles of Tibetan and Han adolescents and (b) to examine the direct and interactive effects of affective empathy, cognitive empathy, and ethnicity on psychosocial adjustment profiles in Chinese adolescents.

Regarding the first goal, due to a dearth of research on psychosocial adjustment profiles of Tibetan and Han adolescents and the exploratory nature of these profiles in the current study, no specific hypothesis about psychosocial profiles is made. However, based on the literature reviewed above (i.e., Zhao et al., 2019), and the potential vulnerabilities of Tibetan adolescents in the process of acculturation, we expected that a lower proportion of Tibetan adolescents may report an adequate adaptation profile but a higher proportion internalizing and/or externalizing problem profiles, as compared with Han adolescents. In terms of the second goal, given the theoretical and empirical considerations, we assumed that a higher proportion of adolescents with higher levels of empathy sort into an adequate adaptation profile, and a lower proportion sort into internalizing and/or externalizing problem profiles, and this association is stronger for Tibetan than Han adolescents: the hypothesized stronger association should depend on Tibetans’ spiritual beliefs (O’Connor et al., 2015). Moreover, prior research showed that sociodemographic characteristics (i.e., gender and age) are potentially related to our dependent variables. For example, female adolescents report higher levels of internalizing and externalizing problem behaviors (Ling et al., 2016) and lower levels of subjective well-being than males (Ma et al., 2015). Age is positively linked to problem behaviors (Zhu et al., 2016), and negatively associated with subjective well-being (Leung et al., 2010; Liu et al., 2016) in Chinese adolescents. Thus, this study regarded age and gender as potential covariates.

## Materials and Methods

### Participants and Procedure

After confirming the ethical approval from the Northwest Minzu University, the authors contacted the public middle and high schools in north mainland China. These schools were close to Tibetan settlements in this region, and Tibetan and Han adolescents were the main ethnicities in these public schools. After receiving the approval from the school principals, written informed consent was sent to each adolescent, who was asked to bring this material back home for parent’s signature. In the meanwhile, each adolescent was asked whether he or she was willing to participate in this project. Only on condition that we obtained both written and vocal agreements from parents and adolescents, would participants be allowed to recruit in this study.

In total, 306 Tibetan adolescents (66.3% girls) and 321 Han adolescents (55.1% of girls) were recruited in the current study. The mean ages were 16.44 (*SD* = 1.74) and 15.69 (*SD* = 1.63) years for Tibetan and Han adolescents, respectively, with age ranging from 12 to 18 years.

Since the medium of the curriculum at school was Mandarin, all the adolescents were asked to complete the measurements in simplified Chinese. Before the assessment, research assistants who were familiar with both languages examined the availability of Mandarin in Tibetan adolescents to ensure the applicability of these measurements. Suggested by prior research (Lan et al., 2019a), we randomly selected 20% items from the measurement battery and asked the participants who were involved in this study to translate and back-translate in front of research assistants. This procedure was in accordance with the standardized procedure from a cross-cultural perspective (Van de Vijver and Leung, 1997). Therefore, all the involved Tibetan adolescents were ensured that they did not have difficulties with understanding the assessment battery in simplified Chinese.

During school hours, research assistants provided standardized instructions, and then adolescents were asked to fill in a set of questionnaires in the classroom. Since our survey battery included a number of questionnaires that were asked to fill in by respondents, we divided the whole assessment into two sessions to avoid the potential burdens and fatigues in adolescents. Each of the session approximately took a regular class hour in China (i.e., 45 min).

### Measures

#### Empathy

Affective empathy and cognitive empathy were assessed by two subscales – empathic concern and perspective taking – of the Interpersonal Reactivity Index (IRI; Davis, 1983). These scales have been validated in Chinese populations by Siu and Shek (2005). Sample items include, “When I see someone being taken advantage of, I feel kind of protective towards them” (empathic concern, 7 items) and “When I am upset at someone, I usually try to ‘put myself in his shoes’ for a while” (perspective taking, 7 items). Participants were asked to rate each item on a 5-point scale ranging from 1 (*does not describe me at all*) to 5 (*describes me very well*). Prior research showed good internal consistency for this scale (Siu and Shek, 2005). In this study, Cronbach’s alpha were 0.69 and 0.70 for affective empathy in Tibetan and Han adolescents, respectively, and 0.70 and 0.71 for cognitive empathy in Tibetan and Han adolescents, respectively.

#### Psychosocial Adjustment Indicators

Depression was measured by the 20-item Center for Epidemiological Studies – Depression Scale (Radloff, 1977). This scale was validated in Chinese populations by Boey (1999), showing adequate properties. Participants were asked how often he or she experienced specific depressive symptoms during the past week based on a 4-point Likert scale ranging from 0 (*rarely*) to 3 (*sometimes*). One of the examples is “I was bothered by things that usually do not bother me.” A mean score involving all the items was calculated, with higher values indicating severe depressive symptoms. Prior research has shown good internal consistency of this scale in Chinese populations (Lan et al., 2019c). In this study, Cronbach’s alpha were 0.83 and 0.85 in Tibetan and Han adolescents, respectively.

Loneliness was assessed by the 16-item Chinese adaption of the Loneliness scale (Asher et al., 2006; Chen L. et al., 2014), showing proper reliability and validity in Chinese youth. One of the examples is “I do not have anybody to play with at school.” Participants were asked to rate each item based on a 5-point scale ranging from 1(*not at all true*) to 5(*always true*). The average score of their responses was calculated, with higher scores indicating greater loneliness. Previous study has signified good internal consistency of this scale in Chinese adolescents (Lan and Moscardino, 2019). In this study, Cronbach’s alpha were 0.94 and 0.95 in Tibetan and Han adolescents, respectively.

Life satisfaction was assessed by the Chinese translation of The Satisfaction With Life Scale (SWLS; Diener et al., 1985), which contains 5 items. One of the examples is “In most ways, my life is close to my ideal.” Respondents were asked to indicate the extent to which he or she agreed with each item on a 7-point Likert scale ranging from 1 (*strongly disagree*) to 7 (*strongly agree*). All the items were averaged to yield a composite score, with high values indicating higher levels of life satisfaction. According to prior research, SWLS has demonstrated good internal consistency in Chinese (Shek, 2004). In this study, Cronbach’s alpha were 0.81 and 0.86 in Tibetan and Han adolescents, respectively.

Positive and negative affect were measured by the 14-item Affect Balance Scale (ABS; Bradburn, 1969), which has been validated in Chinese populations by Chen and Zhang (2004). ABS consists of two facets: positive affect (8 items; I feel particularly excited or interested in something) and negative affect (6 items; I feel so restless that I cannot sit long in a chair). Participants were asked to rate each item according to the frequency of each experience in their daily lives on a 4-point scale ranging from 1(*never*) to 4 (*always*). The average score for each dimension was calculated, with a higher score indicating a higher level of positive or negative affect. Based on previous study in Chinese adolescents, this scale showed good properties (Yang et al., 2017). In the present study, Cronbach’s alpha for positive affect were 0.90 and 0.91 in Tibetan and Han adolescents, respectively. In terms of negative affect, Cronbach’s alpha were 0.80 and 0.85, respectively.

Prosocial behavior was measured by the 15-item Chinese version of the Prosocial Behavior Scale (PBS), which was originally developed for Chinese adolescents (Yang et al., 2016). One of the examples is “I like participating in social activities for the public good.” Participants were asked to rate each item from 1 (*definitely does not apply to me*) to 7 (*definitely applies to me*) on the Likert scale. The average score of 15 items was calculated, with higher scores indicating a higher level of prosocial behavior. Prior research has illustrated good internal consistency of this scale in Chinese adolescents (Yang et al., 2017). In the present study, Cronbach’s alpha were 0.92 and 0.94 in Tibetan and Han adolescents, respectively.

Antisocial behavior was measured by the two subscales (i.e., aggressive behavior and delinquency) of Chinese adaption of the Child Behavior Checklist (Achenbach, 1991; Li et al., 2009), which consists of 9 items. One of the examples is “I destroy my own things.” Participants were asked to rate each item about how often they experience specific aggressive and delinquent behaviors during the past week based on 3-point Likert-type scale ranging from 0 (*not true*) to 2 (*certainly true*). All the items were averaged, with higher scores indicating severe antisocial behavior. Previous studies have shown good internal consistency of this scale in Chinese adolescents (e.g., Lan et al., 2019b). In the current study, Cronbach’s alpha were 0.89 and 0.91 in Tibetan and Han adolescents, respectively.

#### Data Analyses

Data analyses were performed in SPSS 21.0 (IBM Corp, 2012), Mplus 7.0 (Muthén and Muthén, 2012), and Jamovi 0.9.6.9 (The Jamovi Project, 2019). No influential cases were excluded due to high rates of missing data (more than 20%). To investigate the impact of missing data (less than 20%), Little’s Missing Completely at Random (MCAR) test was conducted. Results supported the MCAR assumption, χ*^2^*(129) = 149.25*, p* = 0.07. Therefore, missing data could be safely estimated in SPSS with an expectation-maximization algorithm.

First, means and standard deviations for study variables and intercorrelations were calculated by separating Tibetan and Han adolescents.

Second, a latent profile analysis was conducted in Mplus 8.0 (Muthén and Muthén, 2012) to identify potential psychosocial adjustment profiles. Suggested by prior research (e.g., Zhao et al., 2019), 1- to 5-profile solutions were evaluated and compared based on fit statistics, interpretability, and theoretical considerations to determine the optimal number of latent profiles. An optimal model fit was selected in the context of lower Akaike information criterion (AIC) values, Bayesian information criterion (BIC) values, and adjusted Bayesian information criterion (aBIC) values, as well as higher entropy, a significant bootstrapped likelihood ratio test (BLRT), and a significant Lo-Mendell-Rubin adjusted likelihood ratio test (LMR-LRT). Moreover, to determine whether these profiles significantly differed on the selected psychosocial indicators, multivariate analysis of variance (MANOVA) and the follow-up *post hoc* test was conducted.

Third, a multiple multinomial logistic regression using the package GAMLj in Jamovi (Gallucci, 2019) was implemented to assess the direct and interactive effects of affect empathy, cognitive empathy, and ethnicity on psychosocial adjustment profiles. In this analysis, categorical variables (i.e., gender and ethnicity) were dummy coded and continuous variables (i.e., age, affective empathy, and cognitive empathy) were mean-centered prior to creating and entering the interaction terms into the equation, as suggested by prior research (Ang and Goh, 2010). Moreover, to understand the nature of the interactions, simple slope analysis was performed accordingly.

## Results

### Descriptive Statistics

Means and standard deviations for study variables and bivariate correlations, separately for Tibetan and Han adolescents, are shown in Table 1.

**TABLE 1:** Descriptive statistics and bivariate correlations of study variables for Tibetan and Han adolescents.

	**Tibetan**		**Han**										
	***M***	***SD***	***M***	***SD***	**1**	**2**	**3**	**4**	**5**	**6**	**7**	**8**	**9**
1. AE	3.22	0.48	3.11	0.58	–	0.67^∗∗∗^	0.27^∗∗∗^	0.13^∗^	–0.03	–0.01	0.15^∗∗^	0.05	0.10
2. CE	3.29	0.51	3.19	0.60	0.48^∗∗∗^	–	0.11	–0.09	0.14^∗^	0.16^∗∗^	–0.06	0.18^∗∗^	–0.07
3. Depression	1.08	0.40	0.96	0.44	0.15^∗∗^	0.05	–	0.40^∗∗∗^	–0.27^∗∗∗^	–0.34^∗∗∗^	0.54^∗∗∗^	–0.18^∗∗^	0.33^∗∗∗^
4. Loneliness	2.17	0.71	2.25	0.74	–0.02	–0.11	0.57^∗∗∗^	–	–0.32^∗∗∗^	–0.36^∗∗∗^	0.40^∗∗∗^	–0.37^∗∗∗^	0.28^∗∗∗^
5. Life satisfaction	3.82	1.10	4.00	1.18	–0.05	0.06	–0.41^∗∗∗^	–0.48^∗∗∗^	–	0.45^∗∗∗^	–0.39^∗∗∗^	0.29^∗∗∗^	–0.19^∗∗^
6. Positive affect	2.66	0.61	2.74	0.63	0.14^∗^	0.18^∗∗^	–0.35^∗∗∗^	–0.47^∗∗∗^	0.49^∗∗∗^	–	–0.46^∗∗∗^	0.34^∗∗∗^	–0.23^∗∗∗^
7. Negative affect	2.18	0.53	2.09	0.59	0.04	–0.03	0.61^∗∗∗^	0.46^∗∗∗^	–0.40^∗∗∗^	–0.38^∗∗∗^	–	–0.15^∗∗^	0.28^∗∗∗^
8. Prosocial behavior	5.25	0.91	5.17	1.05	0.19^∗∗^	0.40^∗∗∗^	–0.28^∗∗∗^	–0.46^∗∗∗^	0.32^∗∗∗^	0.33^∗∗∗^	–0.31^∗∗∗^	–	–0.30^∗∗∗^
9. Antisocial behavior	0.15	0.24	0.13	0.23	0.02	–0.15^∗∗^	0.32^∗∗∗^	0.24^∗∗∗^	–0.18^∗∗^	–0.10	0.19^∗∗^	–0.33^∗∗∗^	–

*Correlation coefficients displayed below the diagonal are for Tibetan adolescents (*n* = 306), above for Han adolescents (*n* = 321). AE = affective empathy; CE = cognitive empathy. ^∗^*p* < 0.05, ^∗∗^*p* < 0.01, ^∗∗∗^*p* < 0.001.*

As shown in Table 1, for Tibetan adolescents, affective empathy and cognitive empathy were each significantly and positively related to positive affect and prosocial behavior; affective empathy was positively associated with depression; cognitive empathy was negatively associated with antisocial behavior. For Han adolescents, affective empathy was positively associated with depression, loneliness, and negative affect; cognitive empathy was positively associated with life satisfaction, positive affect, and prosocial behavior.

### Latent Profile Analysis

A latent profile analysis was used to identify potential psychosocial adjustment profiles. The model fit indices are reported in Table 2.

**TABLE 2:** The goodness of fit indices for different latent psychosocial adjustment profiles.

	**AIC**	**BIC**	**aBIC**	**Entropy**	**LMR-LRT**	**BLRT**	**Smallest profiles (%)**
1-Profile	9007.69	9069.86	9025.42	–	–	–	–
2-Profile	8309.52	8407.22	8337.37	0.72	700.57^∗^	714.17^∗^	39.7
3-Profile	8003.19	8136.42	8041.17	0.80	316.19^∗^	322.33^∗^	8.1
4-Profile	7811.75	7980.50	7859.86	0.82	203.49	207.44	6.4
**5-Profile**	**7661.43**	**7865.71**	**7719.68**	**0.84**	**163.15^∗∗^**	**166.32^∗∗^**	**3.7**

**N* = 627. AIC = akaike information criteria; BIC = bayesian information; aBIC = adjusted bayesian information; LMR-LRT = lo-mendell-rubin adjusted likelihood ratio test; BLRT = bootstrapped likelihood ratio test. ^∗^*p* < 0.05, ^∗∗^*p* < 0.01. The bold font refers to the optimal solution.*

As shown in Table 2, the LMR-LRT and BLRT values were significant for the 2-, 3-, and 5-profile solutions. Compared to the 2- and 3- profile options, the 5-profile option showed lower levels of AIC, BIC, and aBIC, and higher entropy. Thus, the results supported a 5-profile as an optimal solution.

**FIGURE 1:**
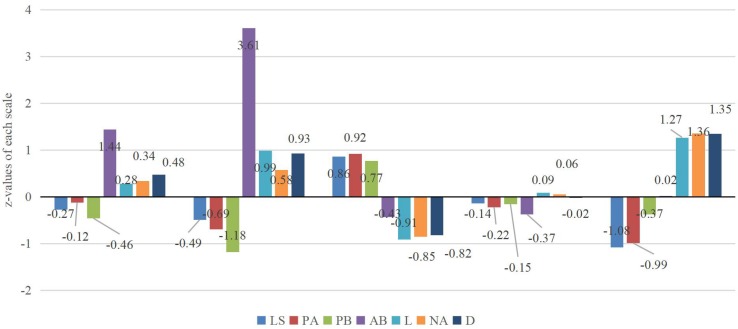
Five psychosocial adjustment profiles based on *z*-values of each scale. *N* = 627. LS = life satisfaction; PA = positive affect; PB = prosocial behavior; AB = antisocial behavior; L = loneliness; NA = negative affect; D = depression. The profiles from left to right are maladapters, externalizing problem individuals, adapters, moderates, and internalizing problem individuals, respectively.

A visualized distribution pertaining to these five profiles is depicted in Figure 1. These profiles were interpreted based on pertinent empirical research (e.g., Zhao et al., 2019). In sum, these profiles involved: (a) maladapters (*n* = 70; 48.5% of Tibetan adolescents) were described by moderate-low levels of positive indicators (i.e., life satisfaction, positive affect, and prosocial behavior) and moderate-high levels of negative indicators (e.g., depression, loneliness, negative affect, and antisocial behavior); (b) externalizing problem individuals (*n* = 23; 56.5% of Tibetan adolescents) were mainly characterized by the highest levels of externalizing problem behavior (i.e., antisocial behavior) and the lowest levels of prosocial behavior; (c) adapters (*n* = 169; 43.7% of Tibetan adolescents) were described by the highest levels of positive indicators (i.e., life satisfaction, positive affect, and prosocial behavior) and the lowest levels of negative indicators (i.e., depression, loneliness, negative affect, and antisocial behavior); (d) moderates (*n* = 299; 58.3% of Tibetan adolescents) were described by moderate levels of all selected indicators; and (e) internalizing problem individuals (*n* = 66; 51.5% of Tibetan adolescents) were mainly characterized by the highest levels of depression, loneliness, and negative affect.

Moreover, mean differences in study variables among psychosocial adjustment profiles are reported in Table 3. As shown in Table 3, MANOVA demonstrated that these five profiles showed significant differences in all psychosocial indicators: depression (*F* (4, 622) = 118.33, *p* < 0.001, partial η^2^ = 0.43), loneliness (*F* (4, 622) = 124.45, *p* < 0.001, partial η^2^ = 0.45), life satisfaction (*F* (4, 622) = 83.81, *p* < 0.001, partial η^2^ = 0.35), positive affect (*F* (4, 622) = 92.10, *p* < 0.001, partial η^2^ = 0.37), negative affect (*F* (4, 622) = 111.47, *p* < 0.001, partial η^2^ = 0.42), prosocial behavior (*F* (4, 622) = 54.37, *p* < 0.001, partial η^2^ = 0.26), and antisocial behavior (*F* (4, 622) = 738.64, *p* < 0.001, partial η^2^ = 0.83). Suggested by prior research (e.g., Datu and Fong, 2018), a *post hoc* test (i.e., Bonferroni method) was used to ascertain specific pair-wise comparisons in all psychosocial indicators. Results showed that significant differences were found for most pair-wise comparisons except for several non-significant differences in these indicators (see details in the footnote of Table 3).

**TABLE 3:** Mean differences in study variables among psychosocial adjustment profiles.

	**1. Maladapters**			**2. Externalizing**			**3. Adapters**			**4. Moderates**			**5. Internalizing**					
	***M***	***SD***	**Range**	***M***	***SD***	**Range**	***M***	***SD***	**Range**	***M***	***SD***	**Range**	***M***	***SD***	**Range**	***F***	**η^2^**	**PH**
D	2.73	0.82	0–2	3.15	1.01	0–2	1.50	0.63	0–1	2.26	0.71	0–2	3.56	0.71	1–2	118.33^∗∗∗^	0.43	52143
L	2.41	0.68	1–4	2.94	0.61	1–4	1.55	0.47	1–4	2.28	0.53	1–4	3.13	0.57	1–5	124.45^∗∗∗^	0.45	52143
LS	3.60	0.93	1–6	3.35	1.20	1–6	4.90	1.01	2–7	3.75	0.84	1–6	2.67	0.95	1–6	83.81^∗∗∗^	0.35	34125
PA	2.63	0.50	1–4	2.28	0.66	1–4	3.27	0.47	2–4	2.57	0.49	1–4	2.08	0.49	1–3	92.10^∗∗∗^	0.37	34125
NA	2.32	0.49	1–4	2.46	0.60	1–4	1.66	0.48	1–4	2.17	0.36	1–3	2.90	0.43	2–4	111.47^∗∗∗^	0.42	52143
PB	4.75	0.91	2–7	4.04	0.71	3–6	5.96	0.76	4–7	5.06	0.91	1–7	4.84	0.76	3–6	54.37^∗∗∗^	0.26	34152
AB	0.48	0.12	0–1	0.99	0.20	0–1	0.04	0.09	0–1	0.06	0.08	0–1	0.15	0.12	0–1	738.64^∗∗∗^	0.83	21543

**N* = 627. D = depression; L = loneliness; LS = life satisfaction; PA = positive affect; NA = negative affect; PB = prosocial behavior; AB = antisocial behavior. PH = *post hoc*, the numbers under PH represent different psychosocial adjustment profiles followed by a descend order. No significant pair-wise comparisons are 1 vs. 2 and 2 vs. 5 in D; 1 vs. 4 and 2 vs. 5 in L; 1 vs. 2, 1 vs. 4, and 2 vs. 4 in LS; 1 vs. 4, 2 vs. 4, and 2 vs. 5 in PA; 1 vs. 2 and 1 vs. 4 in NA; 1 vs. 4, 1 vs. 5, and 4 vs. 5 in PB; and 3 vs. 4 in AB. ^∗∗∗^*p* < 0.001.*

### Multiple Multinomial Analysis

First, the omnibus Chi-square tests indicated that this model explained 7.57% variance of psychosocial adjustment profiles. As reported in Table 4, affective empathy, cognitive empathy, gender, age, and the interaction term between ethnicity and affective empathy significantly explained more of the variance in psychosocial adjustment profiles, but no significant effects were found in terms of ethnicity and the interactive term between ethnicity and cognitive empathy.

**TABLE 4:** The omnibus Chi-square tests.

	***χ*^2^**	***df***	***p***
Gender	23.734	4	<0.001
Age	21.744	4	<0.001
Ethnicity	4.194	4	0.38
Affective empathy	44.384	4	<0.001
Cognitive empathy	35.474	4	<0.001
Ethnicity × affective empathy	12.144	4	0.02
Ethnicity × cognitive empathy	6.154	4	0.19

**N* = 627.*

Next, a multiple multinomial analysis was used to examine the contributions of affective empathy, cognitive empathy, ethnicity (Tibetan vs. Han), potential covariates (i.e., age and gender), and two-way interaction terms between empathy and ethnicity to psychosocial adjustment profiles. In this analysis, we compared all of these psychosocial adjustment profiles with each other. In total, ten times of the profiles contrast was made, and an intact report could be found in the Supplementary Materials. For the sake of brevity, we reported significant results only in Table 5.

**TABLE 5:** Multiple multinomial regression analysis predicting psychosocial adjustment profiles from empathy, ethnicity, sociodemographic characteristics, and interaction effects.

**Profiles contrast**	**Variables**	**Logit**	**SE**	***p***	**Odds ratio**	**95% CI**	
2 vs. 1	Gender^a^	–1.13	0.55	0.04	0.32	–2.21	–0.06
3 vs. 1	AE	–2.65	0.54	<0.001	0.07	–3.71	–1.59
	CE	2.28	0.52	<0.001	9.74	1.26	3.29
4 vs. 1	Gender	0.84	0.28	<0.001	2.31	0.29	1.39
	AE	–1.47	0.49	<0.001	0.23	–2.43	–0.51
	CE	1.01	0.47	0.03	2.76	0.08	1.95
3 vs. 2	Gender	1.59	0.53	<0.001	4.88	0.56	2.61
	Ethnicity^b^	–1.07	0.55	0.05	0.34	–2.14	0.00
	AE	–1.16	0.58	0.05	0.31	–2.30	–0.02
	CE	1.85	0.56	<0.001	6.34	0.74	2.95
	AE × ethnicity	3.25	1.17	0.01	25.76	0.95	5.55
4 vs. 2	Gender	1.97	0.51	<0.001	7.18	0.97	2.98
	Ethnicity	–1.04	0.54	0.05	0.35	–2.09	0.01
	Age	0.44	0.14	<0.001	1.55	0.15	0.72
5 vs. 2	Gender	1.50	0.56	0.01	4.48	0.40	2.60
	Age	0.42	0.16	0.01	1.52	0.11	0.73
4 vs. 3	Gender	0.39	0.21	0.07	1.47	–0.03	0.80
	Age	0.22	0.06	<0.001	1.25	0.10	0.34
	AE	0.51	0.26	0.05	1.66	0.00	1.01
	CE	–1.01	0.24	<0.001	0.37	–1.48	–0.53
	AE × ethnicity	–1.35	0.51	0.01	0.26	–2.35	–0.34
5 vs. 3	Age	0.21	0.09	0.02	1.23	0.03	0.38
	AE	1.97	0.39	<0.001	7.20	1.22	2.73
	CE	–1.43	0.37	<0.001	0.24	–2.16	–0.70
	CE X Ethnicity	1.70	0.75	0.02	5.46	0.24	3.16
5 vs. 4	AE	1.46	0.36	<0.001	4.33	0.77	2.16

**N* = 627. Only significant results were presented above, and the intact report could be found in the Supplementary Materials. ^*a*^Coded as 1 = male, 2 = female, ^*b*^coded as 1 = han adolescents, 2 = tibetan adolescents. AE = affective empathy, CE = cognitive empathy. 1 = maladapters, 2 = externalizing problem individuals, 3 = adapters, 4 = moderates, and 5 = internalizing problem individuals.*

As shown in Table 5, in terms of profiles contrast between the maladaptive profile and others, the results found that individuals with low affective empathy and high cognitive empathy were more likely to be a member of adaptive and moderate profiles; males were more likely than females to be a member of the externalizing profile; females were more likely than males to be a member of the moderate profile. Second, regarding profiles contrast between the externalizing profile and others, the results showed that individuals with low affective empathy and high cognitive empathy were more likely to be a member of the adaptive profile; Han adolescents were more likely than Tibetan adolescents to be a member of adaptive and moderate profiles; females were more likely than males to be member of adaptive, moderate, and internalizing profiles; age was positively associated with the possibility of being a member of moderate and internalizing profiles. Third, with respect to profiles contrast between the adaptive profile and others, the result showed that individuals with high affective empathy and low cognitive empathy were more likely to be a member of moderate and internalizing profiles; females were more likely than males to be a member of the moderate profile; age was positively associated with the possibility of being a member of moderate and internalizing profiles. As for profiles contrast between the moderate profile and others, the results showed individuals with high affective empathy were more likely to be a member of the internalizing profile. Apart from these significant findings, the significant interactive terms involved in these analyses were explained in the next paragraph based on the interactive figures and simple slope analysis.

**FIGURE 2:**
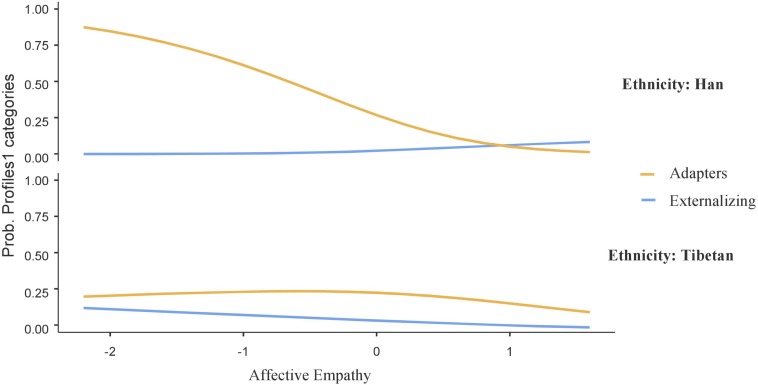
Interaction effect of affective empathy and ethnicity on probability of adaptive and externalizing profiles. *N* = 627.

**FIGURE 3:**
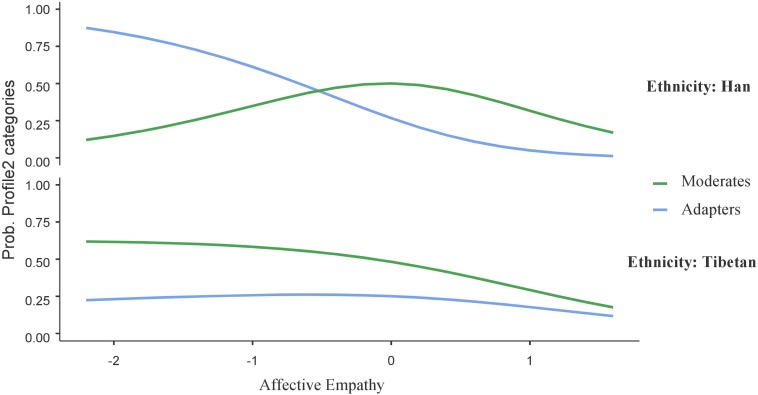
Interaction effect of affective empathy and ethnicity on probability of adaptive and moderate profiles. *N* = 627.

**FIGURE 4:**
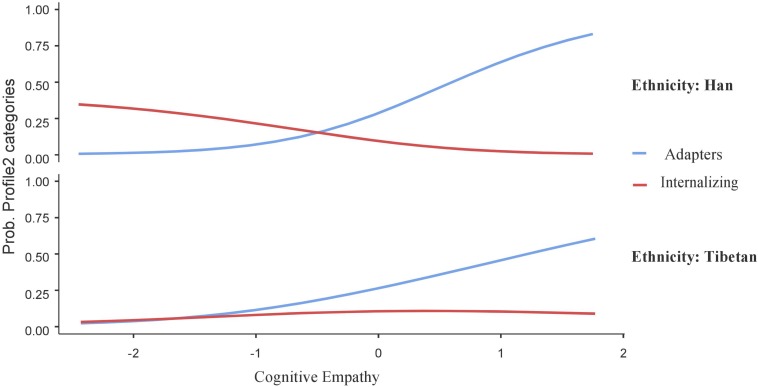
Interaction effect of cognitive empathy and ethnicity on probability of adaptive and internalizing profiles. *N* = 627.

The interactive figures were used to visualize the significant interaction term between affective empathy and ethnicity in the profiles contrast (i.e., maladapters vs. adapters, adapters vs. moderates, and adapters vs. internalizing individuals; see Figures 2–4, respectively).

Aligned with Figure 2, simple slope analysis showed that, in the context of low affective empathy, Han adolescents were more likely to be a member of the adaptive profile (vs. externalizing profile; *B* = −2.78, *SE* = 0.91, *t* = −3.07, *p* = 0.002); however, for Tibetan adolescents, being a member of externalizing or adaptive profiles was regardless of the levels of affective empathy (*B* = 0.46, *SE* = 0.73, *t* = 0.63, *p* = 0.53); meanwhile, in the context of high affective empathy, both Tibetan and Han adolescents did not show significant differences of being a member of externalizing or adaptive profiles.

Moreover, as shown in Figure 3, the results showed that, in the context of low affective empathy, Han adolescents were more likely to be a member of the adaptive profile (vs. moderate profile; *B* = 1.18, *SE* = 0.35, *t* = 3.33, *p* < 0.001); however, for Tibetan adolescents, being a member of moderate or adaptive profiles was regardless of the levels of affective empathy (*B* = −0.16, *SE* = 0.37, *t* = −0.44, *p* = 0.65); meanwhile, in the context of high affective empathy, both Tibetan and Han adolescents did not show significant differences of being a member of adaptive or moderate profiles.

Finally, as shown in Figure 4, the results showed that, in the context of high cognitive empathy, Han adolescents were more likely to be a member of the adaptive profile (vs. internalizing profile; *B* = −2.28, *SE* = 0.54, *t* = −4.21, *p* < 0.001); however, for Tibetan adolescents, being a member of internalizing or adaptive profiles was regardless of the levels of cognitive empathy (*B* = −0.58, *SE* = 0.51, *t* = −1.13, *p* = 0.25); meanwhile, in the context of low cognitive empathy, both Tibetan and Han adolescents did not show significant differences of being a member of adaptive or internalizing profiles.

## Discussion

Given the dearth of research investigating potential psychosocial difficulties of Tibetan adolescents when coping with the mainstream culture, the current study adopted a person-centered approach in identifying psychosocial adjustment profiles of Tibetan adolescents and Han adolescents. Also, guided by a theoretical framework of bilingual-bicultural adaptation and empathy theory, we explored the direct and interactive effects of affective empathy, cognitive empathy, and ethnicity on these psychosocial adjustment profiles. The findings showed five psychosocial adjustment profiles: maladapters, externalizing problem individuals, adapters, moderates, and internalizing problem individuals. Han adolescents were more probable than Tibetan adolescents to be members of adaptive and moderate profiles, less probable to be members of externalizing profile. Moreover, individuals with high affective empathy and low cognitive empathy were more likely to be members of all inadequate profiles (i.e., maladaptive, externalizing, and internalizing) than of adequate profiles (i.e., adaptive and moderate profile). Additionally, the interaction between empathy and ethnicity showed that, in the context of low affective empathy, Han adolescents were likely to be members of the adaptive profile, and less probable to be members of moderate and externalizing profiles; in the context of high cognitive empathy, Han adolescents were likely to be members of the adaptive profile, and less probable to be members of the internalizing profile; however, for Tibetan adolescents, these possibilities had no relation to their levels of affective and cognitive empathy.

Our first objective was to identify psychosocial adjustment profiles in Tibetan and Han adolescents based on a person-centered approach. The findings showed five profiles. The adaptive profile showed the strongest psychosocial adjustment pattern, which was characterized by the highest scores on all positive indicators, and the lowest scores on all negative measures. Moreover, the moderate profile was demonstrated by all moderate levels of indicators. Comparatively speaking, externalizing, internalizing, and maladaptive profiles were outlined by psychosocial adjustment difficulties. Of these inadequate profiles, the externalizing profile was defined by the highest levels of behavioral problems, while the internalizing profile was defined by the highest levels of emotional problems, and the maladaptive profile was characterized by both moderate emotional and behavioral difficulties. These profiles are in accordance with pertinent conceptual and empirical connections between competence and psychopathology in development (Masten and Curtis, 2000; Zhao et al., 2019). However, compared with the research of Zhao et al., the current study replicated previous profiles with the addition of maladaptive and moderate profiles. This may because Zhongyong thinking (the Doctrine of the Mean) is a distinctive and critical philosophical thought in Chinese societies (Yang et al., 2016). Thus, in terms of both adequate and inadequate profiles, there was a portion of adolescents tending to report moderate levels of problems and adjustments.

The second aim of this study was to explore the direct and interactive effects of affective empathy, cognitive empathy, and ethnicity on psychosocial adjustment profiles. Regarding the direct effect of ethnicity, the current study showed that Han adolescents were more likely than Tibetan adolescents to be members of adaptive and moderate profiles, and less probable to be members of the externalizing profile. These findings are in line with previous studies suggesting the vulnerabilities of Tibetan adolescents (Chen X. et al., 2014; Lu et al., 2018). One possible explanation is ascribed to family socioeconomic status. Most Tibetan adolescents grow up in nomadic or farming areas due to Tibet’s unique geographical environment (Yong, 2013), and their family incomes are relatively lower than Han families; by contrast, most Han adolescents are nurtured in industrialized societies. Based on prior research (Bradley and Corwyn, 2002), family socioeconomic status is positively associated with a wide array of health and socioemotional outcomes in adolescence. Therefore, Han adolescents in families with higher socioeconomic status are more likely than Tibetan adolescents to be members of adaptive and moderate profiles. Another possible interpretation is attributable to acculturation difficulties of Tibetan adolescents when coping with the mainstream culture. Prior research indicates that positive acculturation can facilitate psychosocial functioning of multicultural adolescents (Yeh, 2003). However, Tibetan adolescents may experience acculturation difficulties in the process of adjusting to the mainstream Chinese culture due to language barriers and insufficient educational background (Yong, 2013).

With respect to the direct effects of affective empathy and cognitive empathy on psychosocial adjustment profiles, the current study showed that adolescents with high affective empathy and low cognitive empathy are more likely to be members of inadequate profiles (i.e., maladaptive, externalizing, and internalizing profiles) than adequate profiles (i.e., adaptive and moderate profiles). These findings are in accordance with prior research in adolescence indicating that extreme levels of affective empathy may pose a risk for psychological functioning (e.g., depression and anxiety; Tone and Tully, 2014). One possible explanation is that empathic sensitivity can intensify interpersonal guilt in response to stress and others’ distress. This sense of guilt may then increase the risk for maladaptive psychosocial functioning. As for cognitive empathy, findings from prior research are relatively consistent, suggesting that reduced cognitive empathy is positively associated with inadequate behaviors, such as conduct problems, antisocial behavior, and prosocial behavior in adolescence (Crocetti et al., 2016; Gambin and Sharp, 2016).

With regard to covariates, the current study showed that females were more likely to be members of internalizing, adaptive, and moderate profiles, whereas males were more likely to be members of the externalizing problems profile. These findings are aligned with prior research (Leadbeater et al., 1999), suggesting that females tend to display more internalizing symptoms, such as depression and anxiety, and fewer externalizing symptoms, such as aggressive behavior and delinquent behavior, than do males. One possible interpretation is that females tend to have interpersonal vulnerabilities, and individuals with high interpersonal vulnerability are prone to see themselves as helpless and abandoned by others, which in turn increases the risk for internalizing problems. Moreover, females are more likely to regard their parents as being trustworthy and available to protect them from externalizing problems, whereas males may have more opportunities to exercise their autonomy and competence outside their parental supervision (Leadbeater et al., 1999). Due to positive parent-child relationships between female adolescents and their parents, females may be less likely than males to be members of the externalizing profile and more likely to be members of the adaptive profile. Additionally, the current study showed that age was positively related to the possibility of membership in internalizing and moderate profiles. One possible explanation is ascribed to increased stressful life events (e.g., interpersonal problems and academic pressure) as the children grow older (Zhang et al., 2013). Likewise, with the development of cognition, older adolescents may tend to understand positive and negative life events dialectically, and thus they are prone to be neutral in terms of their self-evaluated emotional and behavioral functioning.

Furthermore, the current findings showed the interactive effect between empathy and ethnicity on psychosocial adjustment profiles. To be specific, in the context of low affective empathy and high cognitive empathy, Han adolescents were more likely to be members of the adaptive profile. This is partially in line with prior research suggesting that affective empathy is positively associated with internalizing problems in adolescents (Geng et al., 2012; Gambin and Sharp, 2016), and cognitive empathy is inversely related to externalizing problems (Li et al., 2015). Thus, individuals with low affective empathy and high cognitive empathy are prone to be members of the adaptive profile with lower levels of internalizing and internalizing problems.

Against from related theory and our hypothesis, the current study failed to find the positive effects of affective empathy and cognitive empathy on buffering psychosocial difficulties in Tibetan adolescents. One possible interpretation is ascribed to the empathy scale in the current study. As documented by prior research (Zeng et al., 2016), the self-compassion scale (showing loving-kindness toward all living beings) is not validated in a Buddhist sample in China, indicating that the theoretical concept of self-compassion is not reflected in Chinese language. Notably, self-compassion and empathy are highly correlated characteristics (Wei et al., 2011), and in this study the empathy scale showed relatively low internal consistency in Tibetan adolescents. This may suggest the empathy scale is not psychometrically sound in terms of Tibetan adolescents. However, due to several limitations in the present research (see the next paragraph), the unexpected results should be considered with caution. Future studies are warranted to confirm the current findings by validating the empathy scale in their original language among Tibetan adolescents. Another explanation is the sparse time of Tibetan adolescents involved in spiritual and religious practices. In modern Chinese societies, adolescents commonly experience high levels of pressure from parental expectations to achieve better academic performance and to perform well in the entrance examination to college (Quach et al., 2015). Likewise, Tibetan adolescents may confront several academic difficulties due to language barriers and insufficient education background, as illustrated by prior research (Yong, 2013). Thus, Tibetan adolescents have less time than Han adolescents to be engaged in other activities, such as spiritual and religious practices. As such, the positive effect of empathy on buffering psychosocial difficulties may be weakened.

Based on these findings, the current research may have several theoretical and practical implications. From a theoretical perspective, this study enriches the bilingual-bicultural adaptation and empathy theories, as well as pertinent literature that explains how and why affective empathy, cognitive empathy, and ethnicity are associated with psychosocial adjustment in Tibetan and Han adolescents. Practically speaking, the current study indicates that minimizing affective empathy and promoting cognitive empathy may facilitate Chinese adolescents’ psychosocial functioning. As for the potential vulnerabilities of Tibetan adolescents when coping with the Han culture, other stress-buffering traits, such as grit, should be considered (Lan et al., 2019a).

Despite these theoretical and practical implications, there are several limitations that should be taken into account when interpreting the current findings. First, this study relies on a cross-sectional design, which has less power than a longitudinal design to establish the causality of study variables. Future studies should use a longitudinal design to explore the association between empathy and psychosocial adjustment and to identify the trajectories of psychosocial adjustment in Tibetan and Han adolescents. Second, although the current study divides the assessment into two sessions, the time required to complete the sets of self-reported questionnaires may be excessive for adolescents; as a result, respondents may simply lose interest and not answer questions accurately. Future studies should consider a multi-informant design or a mixed-methods approach to confirm the current findings. Third, the measurement pertaining to empathy shows a relatively lower internal consistency in this study. These coefficients suggest that future studies should consider using a more powerful and indigenous assessment to confirm the current findings. Fourth, although Tibetan and Han adolescents are recruited from the same schools and they have similar social characteristics, this study does not control for socioeconomic status when comparing psychosocial adjustment in the two groups. Future studies should obtain data on this indicator and control for it as well, in order to avoid potential covariates.

## Conclusion

The current study suggests that complex configurations of psychosocial adjustment seem to exist in Tibetan and Han adolescents. Compared with Han adolescents, Tibetan adolescents seem vulnerable in terms of psychosocial functioning. Low affective empathy and high cognitive empathy may strengthen their psychosocial functioning, and the effects of low affective empathy and high cognitive empathy on psychosocial functioning are stronger in Han adolescents.

## Author’s Note

The authors declared that they did not have any ethnical prejudice or ethnocentrism tendencies. All the results and interpretations were based on the current research samples.

## Data Availability

The datasets generated for this study are available on request to the corresponding author.

## Ethics Statement

Prior to the data collection, ethics approval was granted by the Northwest Minzu University. All the participants offered the informed consent and agreed to participate in this study.

## Author Contributions

CM conceived and drafted the manuscript. YM and YW were the principal investigators of this study. XL performed the statistical analyses and critically revised the manuscript. All authors read and approved the final draft of the manuscript.

## Conflict of Interest Statement

The authors declare that the research was conducted in the absence of any commercial or financial relationships that could be construed as a potential conflict of interest.
