# Supplementary material for: Empathy and Psychosocial Adjustment in Tibetan and Han Adolescents: A Person-Centered Approach
Source: Front Psychol. 2019 Aug 13;10:1896. doi: 10.3389/fpsyg.2019.01896 (PMC6700380; doi:10.3389/fpsyg.2019.01896)
Supplement: Supplementary file 1 [file Table_1.DOCX]

**Supplementary Material**

Table 1

*Multiple multinomial regression analysis predicting psychosocial adjustment profiles from empathy, ethnicity, sociodemographic characteristics, and interaction effects*

| Profiles contrast | Variables | Logit | SE | *p* | Odds ratio | 95% CI | |
| --- | --- | --- | --- | --- | --- | --- | --- |
| 2 vs. 1 | **Gender ^a^** | **-1.13** | **0.55** | **0.04** | **0.32** | **-2.21** | **-0.06** |
|  | Ethnicity ^b^ | 0.97 | 0.59 | 0.10 | 2.64 | -0.18 | 2.12 |
|  | Age | -0.30 | 0.16 | 0.06 | 0.74 | -0.60 | 0.01 |
|  | AE | 0.13 | 0.93 | 0.89 | 1.14 | -1.69 | 1.96 |
|  | CE | -0.24 | 0.93 | 0.79 | 0.78 | -2.07 | 1.58 |
|  | AE X Ethnicity | -1.84 | 1.21 | 0.13 | 0.16 | -4.22 | 0.54 |
|  | CE X Ethnicity | 1.04 | 1.17 | 0.37 | 2.82 | -1.25 | 3.32 |
| 3 vs. 1 | Gender | 0.45 | 0.30 | 0.14 | 1.57 | -0.15 | 1.05 |
|  | Ethnicity | -0.09 | 0.34 | 0.78 | 0.91 | -0.75 | 0.56 |
|  | Age | -0.08 | 0.09 | 0.36 | 0.92 | -0.26 | 0.09 |
|  | **AE** | **-2.65** | **0.54** | **< .001** | **0.07** | **-3.71** | **-1.59** |
|  | **CE** | **2.28** | **0.52** | **< .001** | **9.74** | **1.26** | **3.29** |
|  | AE X Ethnicity | 1.41 | 0.74 | 0.06 | 4.09 | -0.05 | 2.87 |
|  | CE X Ethnicity | -0.31 | 0.71 | 0.67 | 0.74 | -1.71 | 1.09 |
| 4 vs. 1 | **Gender** | **0.84** | **0.28** | **< .001** | **2.31** | **0.29** | **1.39** |
|  | Ethnicity | -0.07 | 0.31 | 0.82 | 0.93 | -0.68 | 0.54 |
|  | Age | 0.14 | 0.08 | 0.10 | 1.15 | -0.02 | 0.30 |
|  | **AE** | **-1.47** | **0.49** | **< .001** | **0.23** | **-2.43** | **-0.51** |
|  | **CE** | **1.01** | **0.47** | **0.03** | **2.76** | **0.08** | **1.95** |
|  | AE X Ethnicity | 0.06 | 0.68 | 0.93 | 1.06 | -1.26 | 1.39 |
|  | CE X Ethnicity | 0.21 | 0.64 | 0.75 | 1.23 | -1.05 | 1.46 |
| 5 vs. 1 | Gender | 0.36 | 0.35 | 0.30 | 1.44 | -0.33 | 1.06 |
|  | Ethnicity | -0.03 | 0.41 | 0.94 | 0.97 | -0.83 | 0.78 |
|  | Age | 0.12 | 0.10 | 0.24 | 1.13 | -0.08 | 0.33 |
|  | AE | 0.06 | 0.60 | 0.92 | 1.06 | -1.12 | 1.25 |
|  | CE | 0.00 | 0.59 | 1.00 | 1.00 | -1.17 | 1.16 |
|  | AE X Ethnicity | -0.07 | 0.84 | 0.94 | 0.93 | -1.72 | 1.58 |
|  | CE X Ethnicity | 1.39 | 0.82 | 0.09 | 4.02 | -0.22 | 3.00 |
| 3 vs. 2 | **Gender** | **1.59** | **0.53** | **< .001** | **4.88** | **0.56** | **2.61** |
|  | **Ethnicity** | **-1.07** | **0.55** | **0.05** | **0.34** | **-2.14** | **0.00** |
|  | Age | 0.21 | 0.15 | 0.15 | 1.24 | -0.07 | 0.50 |
|  | **AE** | **-1.16** | **0.58** | **0.05** | **0.31** | **-2.30** | **-0.02** |
|  | **CE** | **1.85** | **0.56** | **< .001** | **6.34** | **0.74** | **2.95** |
|  | **AE X Ethnicity** | **3.25** | **1.17** | **0.01** | **25.76** | **0.95** | **5.55** |
|  | CE X Ethnicity | -1.34 | 1.13 | 0.23 | 0.26 | -3.56 | 0.87 |
| 4 vs. 2 | **Gender** | **1.97** | **0.51** | **< .001** | **7.18** | **0.97** | **2.98** |
|  | **Ethnicity** | **-1.04** | **0.54** | **0.05** | **0.35** | **-2.09** | **0.01** |
|  | **Age** | **0.44** | **0.14** | **< .001** | **1.55** | **0.15** | **0.72** |
|  | AE | -0.65 | 0.56 | 0.25 | 0.52 | -1.76 | 0.45 |
|  | CE | 0.84 | 0.54 | 0.12 | 2.32 | -0.22 | 1.91 |
|  | AE X Ethnicity | 1.90 | 1.13 | 0.09 | 6.70 | -0.32 | 4.12 |
|  | CE X Ethnicity | -0.83 | 1.09 | 0.45 | 0.44 | -2.96 | 1.30 |
| 5 vs. 2 | **Gender** | **1.50** | **0.56** | **0.01** | **4.48** | **0.40** | **2.60** |
|  | Ethnicity | -1.00 | 0.60 | 0.09 | 0.37 | -2.17 | 0.17 |
|  | **Age** | **0.42** | **0.16** | **0.01** | **1.52** | **0.11** | **0.73** |
|  | AE | 0.81 | 0.62 | 0.19 | 2.26 | -0.40 | 2.03 |
|  | CE | 0.42 | 0.60 | 0.49 | 1.52 | -0.77 | 1.60 |
|  | AE X Ethnicity | 1.77 | 1.25 | 0.16 | 5.88 | -0.67 | 4.22 |
|  | CE X Ethnicity | 0.35 | 1.21 | 0.77 | 1.43 | -2.01 | 2.72 |
| 4 vs. 3 | **Gender** | 0.39 | 0.21 | 0.07 | 1.47 | -0.03 | 0.80 |
|  | Ethnicity | 0.02 | 0.22 | 0.91 | 1.02 | -0.40 | 0.45 |
|  | **Age** | **0.22** | **0.06** | **< .001** | **1.25** | **0.10** | **0.34** |
|  | **AE** | **0.51** | **0.26** | **0.05** | **1.66** | **0.00** | **1.01** |
|  | **CE** | **-1.01** | **0.24** | **< .001** | **0.37** | **-1.48** | **-0.53** |
|  | **AE X Ethnicity** | **-1.35** | **0.51** | **0.01** | **0.26** | **-2.35** | **-0.34** |
|  | CE X Ethnicity | 0.51 | 0.49 | 0.29 | 1.67 | -0.44 | 1.47 |
| 5 vs. 3 | Gender | -0.09 | 0.31 | 0.78 | 0.92 | -0.69 | 0.52 |
|  | Ethnicity | 0.07 | 0.35 | 0.85 | 1.07 | -0.61 | 0.74 |
|  | **Age** | **0.21** | **0.09** | **0.02** | **1.23** | **0.03** | **0.38** |
|  | **AE** | **1.97** | **0.39** | **< .001** | **7.20** | **1.22** | **2.73** |
|  | **CE** | **-1.43** | **0.37** | **< .001** | **0.24** | **-2.16** | **-0.70** |
|  | AE X Ethnicity | -1.48 | 0.77 | 0.06 | 0.23 | -2.99 | 0.03 |
|  | **CE X Ethnicity** | **1.70** | **0.75** | **0.02** | **5.46** | **0.24** | **3.16** |
| 5 vs. 4 | Gender | -0.47 | 0.29 | 0.10 | 0.62 | -1.04 | 0.09 |
|  | Ethnicity | 0.04 | 0.32 | 0.90 | 1.04 | -0.59 | 0.67 |
|  | Age | -0.01 | 0.08 | 0.86 | 0.99 | -0.18 | 0.15 |
|  | **AE** | **1.46** | **0.36** | **< .001** | **4.33** | **0.77** | **2.16** |
|  | CE | -0.43 | 0.34 | 0.22 | 0.65 | -1.10 | 0.25 |
|  | AE X Ethnicity | -0.13 | 0.71 | 0.85 | 0.88 | -1.52 | 1.26 |
|  | CE X Ethnicity | 1.18 | 0.69 | 0.08 | 3.27 | -0.16 | 2.53 |

Note. *N* = 627. ^a^ coded as 1 = male, 2 = female, ^b^ coded as 1 = Han adolescents, 2 = Tibetan adolescents. AE = affective empathy, CE = cognitive empathy. 1 = maladapters, 2 = externalizing problem individuals, 3 = adapters, 4= moderates, and 5 = internalizing problem individuals.
